# On the Use of Iron in Tic Douloureux

**Published:** 1823-02

**Authors:** Anthony Todd Thomson


*To B. Hutchinson, Esq. Southwell.*

---

Art. IV.—*On the Use of Iron in Tic Douloureux.* By Anthony Todd Thomson, Esq. Communicated by B. Hutchinson, Esq.

91, *Sloane-street, London; October* 1822.

DEAR SIR,

I regret that my absence from home prevented me from answering your letter at an earlier period; and, now that I am returned, I can communicate much fewer details of the cases in which the carbonate of iron proved so beneficial in tic douloureux, than I am assured their importance demands. Both the instances of which reference is made in the last edition of the London Dispensatory were under my care, as senior surgeon of the Chelsea and Brompton Dispensary; but no particular notes of the symptoms, and the progress of the cure, in either were preserved.

From the books of the Dispensary, however, I have pleasure in being able to inform you, that the most important of these two cases was that of a man named James Crooks, a plasterer, aged 35, of a sallow aspect and debilitated frame. On his admission to the charity, 21st July, 1821, he complained of an excruciating pain, which attacked, in successive twinges, the right side of his face, in the direction of a line drawn from the right cuspidatus of the upper jaw to the internal angle of the eye, thence spreading to the temple, the forehead, and across the bridge of the nose. The pain was in quickly succeeding twinges, and he had never experienced a longer interval than two hours from the moment of its first attack, four months before he applied at the Dispensary. The least exposure to cold air, or the taking cold liquids into the mouth, immediately brought on the attack, or greatly increased its violence when it was already present. The torture which he experienced he described as being almost insupportable; and, as the exacerbations were as frequent and severe in the night as during the day, his life had become burthensome to him. His pulse was quick and small, his tongue white; his bowels were open, and the urine rather abundant and limpid. He stated that he had lost much flesh since the disease came on; and his countenance indicated the greatest anxiety and distress.

On making inquiry into the exciting cause of the symptoms under which he was labouring, he stated that about four months ago he had had the "dog tooth," on the right side of the upper jaw, extracted, on account of its decayed state; and, immediately afterwards,—at least, before he reached his house, which was not a quarter of a mile from the house of the surgeon who drew the tooth,—he felt the pain dart from the socket of the extracted tooth upwards to the eye, and thence branch off, as has been already described; and that it had never shifted its place. He applied next day to the surgeon who had operated on him, for relief, and had been bled, and taken much purgative medicine; but, instead of experiencing any benefit from this plan of treatment, the severity of the pain had been greatly augmented.

On the first view which I took of the case, I was of opinion that the disease was tic douloureux, and I resolved to try the method of treating it with subcarbonate of iron; but I first wished to see what effect narcotics, and particularly belladonna, would have, and therefore directed him to take the following pills for ten or twelve days:

℞. Submur. Hydrargyri, gr. j.
Antimonii Tartarizati, gr. $\frac{1}{4}$.
Opii, gr. jss.
Mica panis, q. s. ut ff. pilula mane noctiq. sumenda.

℞. Ferri Subcarbonatis, gr. xl. viij.

Extracti Belladonnæ, gr. xij.

Confectionis Rosæ, q. s. ut ff. pilulæ duodecim, quarum sumaturi, omni mane et meridie, nec non ij 4ta. P.M. horâ, quotidie.

The larger dose was ordered to be taken at four o'clock, because at this time of the day the pain was always the most severe.

On the 1st of August, he stated that the pain had greatly diminished, and that the intervals were much longer, but that it was extremely severe. During this time the subcarbonate of iron had been increased to twelve grains in each of the forenoon doses, and to twenty-four grains in the afternoon dose; and the belladonna had been also doubled in each dose. Anxious to ascertain whether the benefit already received was to be ascribed to the subcarbonate or to the narcotics, I gave each alone for successive periods of ten days; and, being then convinced of the superior efficacy of the preparation of iron, I resolved to push this to the greatest extent to which my patient could take it, and therefore gradually increased the dose of it, whilst the use of the narcotics was discontinued, until a drachm was taken three times a-day. Under this treatment the pain rapidly diminished, and at the end of six weeks he had been several days altogether free from it. His bowels were rendered very costive, but he experienced no other inconvenience from the use of the remedy.

Having considered that he was perfectly cured, he became very irregular in his attendance at the Dispensary, and I lost sight of him for several weeks; at the end of which, having experienced a return of his complaint, he again applied for relief. He was still, however, very irregular in his attendance, and several months elapsed without any alteration; when, having found that the pain was more severe, and the paroxysms more frequent, in proportion as he neglected his medicine, he again resolved to take the subcarbonate in drachm doses three times a-day. The benefit was soon very conspicuous: in two months he lost every trace of the disease; his countenance regained its colour and cheerfulness of expression; his health become in every respect improved; and, when I last saw him, he had become somewhat corpulent. He was for ten months backwards and forwards at the Dispensary.

The other case was also that of a Dispensary patient, but much less severe than that of Crook. The patient was a female, and began the use of the subcarbonate in doses of a scruple three times a-day, rapidly bringing them up to a drachm. She was discharged cured in less than ten days.

I have given the subcarbonate with equal success in several cases which have occurred in my private practice; but I regret my inability to give you any details of these cases, having preserved no notes of them.

The state of the diseased nerve in this painful affection has not yet been determined; but, from having observed that there was a daily increase of the pain nearly about the same hour, and that some other characteristics of the complaint resemble those of intermittents, I long since entertained the opinion that it was to be cured by tonics. Under the influence of this opinion, I prescribed bark and turpentine, in combination with narcotics; and arsenic, both alone and combined with narcotics, at different times; but without obtaining that permanent benefit which has resulted from the employment of the carbonate of iron. I must confess I have no faith in specifics, and would be much gratified could a rational explanation of the *modus operandi* of the carbonate of iron, in curing this painful and distressing complaint, be advanced; for I feel that the observations I have been able to make are too vague to enable me to form any opinion on the subject.

I remain, Sir, your's faithfully,

ANTHONY TODD THOMSON.

*To B. Hutchinson, Esq. Southwell.*

